# Debt Trajectories of Divorcing Mothers and Fathers: Divorce as a Dynamic Economic Uncoupling

**DOI:** 10.21203/rs.3.rs-10145360/v1

**Published:** 2026-08-28

**Authors:** Trisha Chanda, Alec P. Rhodes, Lawrence M. Berger, Rachel E. Dwyer, Jason N. Houle

**Affiliations:** University of Wisconsin–Madison; Purdue University West Lafayette; University of Wisconsin–Madison; The Ohio State University; Dartmouth College

**Keywords:** divorce, debt, gender

## Abstract

Debt is an increasingly central component of American households’ financial portfolios and is closely associated with marital relationship quality. Yet research on the economic consequences of divorce has rarely examined how individual debt holdings change as couples separate. We combine individual-level monthly credit reports with court records on Wisconsin divorce cases involving parents of minor children to study debt trajectories from two years before to two years after divorce petition filing. We find that debt declines in anticipation of divorce and begins to recover after petition filing for both mothers and fathers, although trajectories vary substantially by debt type and parent gender. Asset-backed debts, especially mortgages and auto loans, show the clearest evidence of anticipatory deleveraging and post-petition recovery, while unsecured debts generally decline or remain stable. Supplementary analyses suggest that reductions in non-mortgage debt are concentrated among parents who exit mortgage debt, consistent with the possibility that asset-backed deleveraging helps reduce other obligations. These findings show that debt is not a uniform marker of economic strain: while parents reduce potentially constraining unsecured debts during divorce, access to asset-backed credit may also serve as a resource for rebuilding financial stability after separation.

## Introduction

Debt is an increasingly central component of household financial portfolios and an important dimension of economic well-being (Dwyer, 2018). A growing body of research links debt to relationship formation, stability, and quality, showing that certain forms of debt are associated with delayed marriage (Addo, 2014; Addo et al., 2019) and higher levels of marital conflict (Bridges & Disney, 2016; Dew, 2007, 2008, 2011). Yet, relatively little is known about how debt changes during relationship dissolution or divorce. Divorce represents a fundamental reorganization of family life in which resources, risks, and obligations that were previously shared must be redistributed across separate households. This reorganization involves not only the division of assets but also the allocation and management of credit. Research on the economic consequences of divorce has focused primarily on income, employment, and wealth (e.g., Boertien and Lersch 2021; De Vaus et al. 2017; Holden and Smock 1991; Kapelle and Baxter 2020; Mortelmans 2020; Thielemans and Mortelmans 2022), while liabilities or debts have received far less attention.

Debt can function both as a resource and as a constraint: it can help individuals smooth consumption or access assets, but it can also burden households through repayment obligations. Asset-backed debt, such as mortgages and vehicle loans, may help buffer the financial toll of divorce by preserving access to housing, transportation, or other resources. At the same time, individuals may take on new debt to establish separate households after divorce increasing their repayment burdens, and property division laws may shape how joint liabilities are allocated between spouses. These dynamics are especially salient for parents. Following separation, parents must continue to meet the costs of raising children, often across two households. Custody arrangements and child support systems shape how these costs are distributed and how resources flow between parents (Røed Bilgrei et al., 2025), with implications for both financial need and access to credit. Parents also tend to carry higher levels of debt than nonparents, reflecting the financial demands of childrearing (Bandelj et al., 2024; Kowalski et al., 2023; Xiao & Yao, 2020). As a result, debt during divorce involves not only individual economic adjustment but also the reconfiguration of interdependent financial responsibilities tied to children.

Prior research has rarely examined how debt evolves for mothers and fathers during the divorce process or how these trajectories differ across types of debt, partly because such analyses require access to individual-level debt data linked to divorce records. Moreover, most studies treat divorce as a discrete event, rather than as a process that unfolds over time. Evidence suggests that economic behaviors begin to shift well before formal separation and continue to evolve afterward (Chanda, 2022; Kapelle & Baxter, 2020), highlighting the need for a dynamic perspective on family change.

In this study, we conceptualize divorce as a process of economic uncoupling, in which couples gradually disentangle their financial lives in anticipation of separation and subsequently adjust to new economic circumstances. We examine how debt holdings evolve over the two years preceding and following divorce separately by parent gender and debt type. Specifically, we ask: (1) How do debt holdings evolve over the course of divorce, including in the pre-divorce, or anticipatory, and post-divorce, or recovery, periods? (2) Do these trajectories differ across specific types of debt, including mortgages, auto loans, and unsecured debts? and (3) How do these patterns differ for mothers and fathers?

This study contributes to research on families and economic well-being in three ways. First, it centers debt as a consequential but understudied dimension of post-divorce economic well-being, particularly for parents, for whom divorce reorganizes both individual finances and continuing obligations to children. Second, it advances a dynamic understanding of divorce by examining debt trajectories before and after the legal dissolution of marriage, thereby capturing both anticipatory financial adjustments and post-divorce recovery. Third, it shows that debt is not a uniform marker of economic strain: asset-backed and unsecured debts may play different roles as families deleverage, access resources, and reorganize their financial lives during divorce.

## Background

### Economic Well-being During the Divorce Process

Married couples typically pool economic resources and share assets and liabilities (Beblo & Beninger, 2017; Huang et al., 2019; Kan & Laurie, 2014). Couples financially invest in marriage-specific capital and enjoy economies of scale from sharing a household (Bryant & Zick, 2006). Individuals undergoing divorce therefore experience an economic shock from the loss of previously shared household resources. This shock has gendered effects on employment, needs-adjusted income, and wealth (Boertien & Lersch, 2021; De Vaus et al., 2017; Holden & Smock, 1991; Kapelle & Baxter, 2020; Mortelmans, 2020; Thielemans & Mortelmans, 2022) and is particularly consequential for parents raising children (Thielemans & Mortelmans, 2022).

Research consistently shows that both men and women experience reductions in household income and increased risks of poverty following divorce, although the consequences for women are typically more severe and long-lasting (Bartfeld, 2000; Bartfeld et al., 2012; Bianchi et al., 1999; Bonnet et al., 2021; Eads & Tach, 2016; Leopold, 2018; McManus & Diprete, 2001; Mortelmans, 2020; Thielemans & Mortelmans, 2022). In contrast to poverty and labor market outcomes, the effect of divorce on wealth is more similar for men and women: while men maintain greater absolute wealth because of greater pre-divorce wealth, men and women are similarly unlikely to return to pre-divorce levels of personal wealth several years after divorce (Boertien & Lersch, 2021; Kapelle & Baxter, 2020; Killewald et al., 2023). These differences have been attributed to the fact that wealth reflects a stock of household resources that is often divided according to legal requirements at divorce, whereas income reflects a flow of resources that responds more readily to changes in employment, household composition, and repartnering (Boertien & Lersch, 2021; Kapelle & Baxter, 2020; Killewald et al., 2023). Debt complicates this distinction because it is a liability within the wealth portfolio, but debt repayment also represents a continuing flow out of the household.

### Debt, Relationship Dynamics, and Divorce

Access to and use of credit are now common components of household financial portfolios in the United States, where the aggregate household debt balance has trended upward since the COVID-19 pandemic and associated recession, reaching $17.7 trillion in the first quarter of 2024 (Munsch, 2024). Borrowing decisions depend on both current liquidity needs and expectations about future repayment capacity. Divorce may reduce economies of scale, increase the costs of maintaining separate households, and alter expected income and expenses, especially for parents who continue to support children across households. These changes may increase demand for credit, but they may also reduce access to credit if lenders perceive greater risk or if individuals lose access to jointly held assets. This suggests that asset-backed and unsecured debts may change differently during divorce: asset-backed debt may reflect the reorganization of housing and transportation needs, while unsecured debt may reflect short-term liquidity constraints or financial strain.

Prior work demonstrates that both the incidence of debt (whether debt is held and which types of debt are held) – particularly unsecured debt – and the balance of debt (dollar amounts held) are associated with relationship status and quality (Addo, 2014; Addo et al., 2019; Addo & Zhang, 2020; Bridges & Disney, 2016; Dew, 2007, 2008, 2011; Eads & Tach, 2016). This body of work primarily focuses on the association of debt with relationship formation, stability, and quality. Addo (2014) and Addo and colleagues (2019) find that credit cards and student loans are associated with delayed marriage among young adults. Within-couple concordance in perceptions about outstanding debt has also been associated with greater marital satisfaction (Addo & Zhang, 2020). Studies of divorce and debt have primarily considered how debt can trigger marital strain or dissolution, showing that unsecured debt is associated with greater marital conflict and divorce, whereas asset-backed debt, particularly mortgage debt, is negatively associated with the likelihood of marital dissolution (Bridges & Disney, 2016; Dew, 2007, 2008, 2011; Eads & Tach, 2016). However, this literature has rarely examined how marital dissolution reshapes debt holdings before and after divorce.

### Debt and the Dynamic Economic Uncoupling of Divorce

Divorcing couples typically separate long before a formal court-ordered divorce judgment is finalized (Kapelle & Baxter, 2020; Kreider & Ellis, 2011). Chanda (2022) demonstrates that anticipatory changes in economic behavior, specifically employment, may occur as early as three years prior to a divorce filing. Because couples often divide households, transition living arrangements, and bear the costs of divorce proceedings before final judgment, the effects of divorce on debt holdings may be anticipatory rather than solely post-divorce. These dynamic elements are unmeasured in most studies of the economic consequences of divorce, which operationalize divorce as an event rather than as a process, with the event of divorce being observed at the time the final judgment of divorce is assigned by the court. Our data allow us to observe both the anticipatory and recovery phases of divorce, providing a distinctive window into economic adjustment through credit and debt. It also allows us to identify the time when the divorce petition is filed with the court which serves as a better proxy for the point of separation of households compared to the date of final judgment.

### Debt in the Gendered Anticipation of and Recovery from Divorce

We conceptualize debt trajectories around divorce as unfolding in two phases: an anticipatory phase before formal divorce and a recovery phase afterward. In the anticipatory phase, debt trajectories are theoretically ambiguous. Figure 1 depicts our conceptual model of the relationship between divorce and debt trajectories. Couples may deleverage by reducing jointly held debt, producing declining balances for both mothers and fathers; engage in debt transfer, with one spouse buying out or assuming debt previously shared by the couple; or pursue debt procurement, with one or both spouses acquiring new debt in their own name to manage the financial costs of separation and household reorganization (Figure 1, Panel A). These trajectories may also diverge within couples because debt acquisition and debt management are themselves gendered within households (Callegari et al., 2020). Mothers may take on new unsecured debt to manage divorce-induced financial strain or prepare for independent household formation, while fathers may be better positioned to retain or acquire asset-backed debt because of their stronger pre-divorce economic circumstances and greater access to credit (Chanda, 2022).

These patterns are likely to vary by debt type. Asset-backed debts, such as mortgages and vehicle loans, enable the purchase of potentially wealth-building assets and may signal financial strength and stability. They are also more likely to be jointly held, or at least taken on as joint investments, even when formally held by one partner. By contrast, unsecured debts, such as credit cards and personal loans, are not tied to assets, often support short-term consumption, and may indicate financial insecurity or stress through both the need to borrow and the burden of repayment. Unsecured debts are therefore commonly treated as indicators of economic hardship and have been linked to poorer family wellbeing, especially among families with children (Berger & Houle, 2019; McCloud & Dwyer, 2011; Sweet, 2021). They are also more likely to be held individually and taken on independently (Brevoort & Kambara, 2017)^1^. Thus, during the anticipatory phase, jointly held or asset-backed debts, such as mortgages and vehicle loans, may be especially susceptible to deleveraging or transfer, while individually held unsecured debts may increase if parents rely on credit to cope with the financial stress of divorce.

In the recovery phase, debt trajectories may follow either a “temporary crisis” or “chronic strain” pattern (Figure 1, Panel B). The temporary crisis model suggests that economic outcomes recover and return to baseline within a few years, while the chronic strain model suggests that recovery is slower and may remain incomplete. Prior research indicates that post-divorce recovery depends in part on whether the outcome is a stock, such as wealth, or a flow, such as income. Studies of wealth often support the chronic strain hypothesis, documenting consequences that persist for 5–15 years after divorce (Kapelle & Baxter, 2020; Leopold, 2018). In contrast, studies of income and earnings find more evidence of temporary crisis and substantial recovery within five years, especially among those employed before divorce, those who repartner, and those whose economic losses are less severe (Andreß et al., 2006; Bayaz-Ozturk et al., 2018; Leopold & Kalmijn, 2024).

Debt does not fit neatly into either category. Although debt is a component of wealth, payments on debt also represent a flow out of the household, and asset-backed debt can generate a stock of resources, such as home equity. Debt is also fungible: asset-backed debt may be converted into resources during economic shocks, while unsecured debt may reflect short-term consumption needs or chronic financial strain. For this reason, post-divorce debt trajectories are theoretically ambiguous. Asset-backed debt may rise as parents establish separate households after divorce, but it may recover more quickly for fathers if fathers retain stronger access to credit and asset ownership. Unsecured debt may increase if parents struggle to repay existing obligations or rely more heavily on credit cards and personal loans during household reorganization. This increase may be especially pronounced for mothers if divorce worsens their financial position owing to their weaker pre-divorce economic situation (Bartfeld, 2000; Holden & Smock, 1991; Leopold, 2018; Smock et al., 2024; Thielemans & Mortelmans, 2022) and greater probabilities of living with children post-divorce (Grall, 2020). At the same time, unsecured debt may decline when debt-related distress helped precipitate divorce or when parents deleverage to avoid constraining obligations after separation. Finally, because fathers tend to have stronger pre-divorce economic circumstances (Chanda, 2022), we expect fathers to be more likely to hold debt and to have larger balances than mothers across most debt categories.

These trajectories may reflect two related mechanisms. First, changes in unsecured debt may capture financial strain, as parents rely on credit cards or personal loans to manage income losses or the costs of establishing separate households. Here we use delinquency as an indicator of debt-related financial strain: delinquency captures accounts on which payments are past due, signaling difficulty meeting repayment obligations. Second, changes in asset-backed debt may reflect shifts in ownership and access to wealth-building resources, as parents sell, retain, refinance, or newly acquire homes and vehicles during the divorce process. Examining these mechanisms helps distinguish whether changes in debt primarily reflect economic vulnerability, the reallocation of jointly held obligations, or differential access to credit and assets after divorce. In interpreting our results, we treat anticipatory declines in debt as consistent with deleveraging or debt transfer, depending on whether declines occur similarly for both parents or differ by gender. Post-petition recovery in asset-backed debt is more consistent with a temporary crisis pattern, whereas sustained declines or widening gender gaps may indicate more persistent constraints in access to credit or asset ownership. For unsecured debt, increases during recovery would be consistent with financial strain or debt procurement, while declines may indicate deleveraging, including cases where debt-related distress contributed to divorce.

### Current Study

We study the relationship between divorce and debt holdings using a unique linkage of individual-monthly data on credit and income for a representative sample of divorce cases involving a minor child that appeared before Wisconsin county courts from 2017–2019. Wisconsin has statistically similar rates of marriage and lower rates of divorce than the national average (6.2% vs. 7.7% in 2018) (US Census Bureau, 2020) and follows national trends in levels of debt holdings (authors’ calculations from Wisconsin population and national-level consumer credit panel data). It is also one of nine U.S. states with community property law, which presumes equal ownership of assets between married couples and equal liability for debts acquired during marriage (Wisconsin Act 186, Property Rights of Married Persons; Marital Property, 1983). This provides a useful legal landscape against which de facto arrangements of debt sharing within couples can be examined.

Our data and design have three major innovations. First, Wisconsin court records provide information on both the date a divorce petition was filed and the date of final judgment or divorce completion — a gap of nine months, on average (see Table 1). This allows us to anchor the divorce process around petition, which more closely approximates the timing of separation than final divorce judgment usually available in survey-based data that rely on measures of divorce completion. Second, the individual-monthly structure of our credit and income data allows us to observe the dynamics of divorcing households over a four-year period and to study debt trajectories across both the anticipatory and recovery phases of divorce. Third, our data allow us to explore mechanisms underlying the trends we uncover in our main analyses, including the potential roles of financial strain through an indicator for delinquency and the deleveraging of asset-backed debts such as mortgages.

### Data and methods

#### Data and sample

We use individual-level proprietary credit data from *The Ohio State University-University of Wisconsin Credit Panel* (OSU-UWCP), linked to *Court Records Data* (CRD) on a sample of over 2,000 divorce cases involving minor children in 21 Wisconsin county courts (including the two largest counties – Milwaukee and Dane), from 2017–2019. The OSU-UWCP is organized as monthly individual-level credit records derived from account-level tradeline information reported to a major credit bureau. For each individual-month, we observe balances by debt category, indicators of delinquency, and credit file characteristics. The data capture mainstream credit accounts reported by banks, mortgage lenders, auto lenders, credit card companies, and other financial institutions, but do not capture informal debts, debts not reported to the bureau, or assets. Although account-level tradelines underlie the panel, our analyses use individual-month measures aggregated by debt type.

The CRD sample is designed to be representative of Wisconsin divorce cases involving minor children filed in the sampled counties during 2017–2019, with sampling weights adjusting for county-level sampling probabilities. The OSU-UWCP includes credit history data from a major credit bureau for all Wisconsin consumers with a credit report, which includes monthly data on mainstream accounts from banks, mortgage companies, credit card companies, and auto lenders from 2015–2021. The CRD includes information on families’ and parents’ characteristics, as well as court-recorded income and child support arrangements at divorce. The CRD sample allows us to focus on a population that involves continuing interdependencies between former spouses through custody, child support, and child-related expenses, making the allocation and management of debt especially consequential.

We link these data to the *Wisconsin Administrative Data Core* (WADC; https://www.irp.wisc.edu/wadc/), a harmonized longitudinal data system of individuals participating in a host of public social welfare programs in Wisconsin. The WADC provides longitudinal information on earnings from jobs covered by Unemployment Insurance, child support payments and receipts, and receipt of a host of social welfare benefits.

Of the 2,339 divorce cases in the CRD from 2017–2019, we are able to link 2,244 mothers (96%) and 2,269 fathers (97%) to the WADC and 2,158 mothers and 2,111 fathers (93% and 90%, respectively, of all eligible divorce cases) to the OSU-UWCP.^2^ We observed mothers and fathers in the OSU-UWCP and WADC for 23 months before and 23 months after the date the divorce petition was filed. We anchor event time at the month of divorce petition filing, which we treat as the beginning of the formal dissolution process and a closer proxy for separation than final judgment. Divorce petition filing is not the exact date of marital separation or household separation. Because some couples separate well before filing, changes observed before petition filing may reflect earlier household separation, relationship deterioration, or financial strain that preceded the formal legal process. We therefore interpret the pre-petition period as the anticipatory phase of the dissolution process rather than as a period in which couples are necessarily still co-residing. We restrict our sample to cases in which we have complete data on both spouses from the same divorce case, retaining 1,834 of all eligible divorce cases and creating a balanced panel of 86,198 person-month observations, separately for mothers and fathers. We weight all our analyses to adjust for variation in sampling rates across Wisconsin counties included in the CRD.

### Measures

We use information on monthly debt balances in the OSU-UWCP to construct the following measures.

#### Debt holding.

We measure debt holdings for overall (total) debt and separately for each debt category. We classify mortgage and auto debt as asset-backed debt and credit card and personal loan debt^3^ as unsecured debt. We operationalize debt holdings in two ways: a) *debt incidence:* a monthly indicator for a positive balance (> $0), signifying the extensive margin of debt; b) *debt balance:* a continuous monthly dollar amount of balances. Debt balances include zero balances and are adjusted to 2018 dollars, allowing us to capture debt exposure for the full sample rather than conditioning on individuals with positive balances.

Because credit report data record balances at the individual level, we observe debts reported on each parent’s credit file but do not directly observe legal responsibility for repayment, payment behavior, or whether a debt was jointly incurred during marriage. For measurement purposes, we therefore attribute balances appearing on an individual’s credit report to that individual. The exception is when both spouses in the same divorce case have exactly matching balances within a debt category in the same month; in these cases, we treat the debt as jointly held and assign half of the balance to each spouse to avoid double-counting the same liability. This is a conservative approach to identifying joint debt, as some jointly held debts may not appear as exactly equal across spouses’ credit reports because of reporting timing, fees, interest, or other account-level differences, and the credit data do not allow us to definitively identify legal joint ownership, practical repayment responsibility, or whether a balance was incurred for joint household purposes. We therefore cannot directly test whether the distinctive trajectories of mortgage and auto debt are caused by joint ownership. Instead, we interpret the debt-type differences as consistent with the fact that asset-backed debts are more likely to be jointly held or jointly used household investments. Appendix Table A1 reports the number of observations affected by this assumption and presents sensitivity checks that relax the exact-match rule.

#### Covariates.

We control for age, which may be associated with debt holdings over the life course. We also control for monthly earnings, monthly indicators for any child support paid and any child support received, Temporary Assistance for Needy Families (TANF), and UI benefit receipt. These time-varying economic measures capture contemporaneous financial resources that may shape both parents’ need to borrow and their ability to access or repay debt during the dissolution process. We recognize that these covariates may also lie on the pathway between divorce and debt; accordingly, we estimate supplemental models excluding earnings, child support, TANF, and UI receipt. Results are substantively unchanged, suggesting that the observed debt trajectories are not driven by conditioning on these monthly economic circumstances. All covariates are drawn from the WADC and are measured monthly, with the exception of earnings, which is measured quarterly. We divide quarterly earnings by three to convert to monthly estimates and adjust all dollar values to 2018 dollars. We include an indicator for zero earnings in a given month and another indicator for missing UI earnings information. Finally, we include year-month fixed effects in all models to account for period dynamics.

### Analytical approach

We estimate fixed-effects OLS regressions^4^ predicting debt incidence and (separately) balance in the months since dissolution, stratified by gender:

Eq. (1) $$y_{it}=\beta_{1}{\mathrm{MonthsSinceDissolution}}_{it}+X_{it}^{\prime }\gamma +\delta_{t}+\mu_{i}+\epsilon_{it}$$

where $y_{it}$ represents debt incidence and balance for individual i at time t; $\alpha_{i}$ is the individual level fixed effect, ${\mathrm{MonthsSinceDissolution}}_{it}$ is represented by a series of event-time indicators for each month from 23 months before to 23 months after petition filing, with the month before petition filing^5^ as the reference category for individual i at time t; $X_{it}$ is a vector of individual-level time-varying covariates; $\delta_{t}$ signifies year-month fixed effects; and $\mu_{i}$ denotes time-invariant characteristics. Our primary coefficients of interest are ${\mathrm{MonthsSinceDissolution}}_{it}$, which captures debt trajectories over the divorce process relative to one month preceding the divorce petition filing. For debt incidence, these models are linear probability models. Because debt balances are right-skewed and include a large number of zeros, we interpret balance models as descriptive estimates of average debt exposure in the full sample rather than as estimates of conditional balances among debt holders. We examine debt incidence and balances as complementary outcomes: incidence models capture movement onto or off a debt margin, while balance models capture changes in the total amount of debt reported, including changes driven by entry into or exit from debt. We estimate balance models in levels to preserve the interpretation of debt as a dollar-denominated component of the broader financial portfolio during divorce and to avoid selecting only on individuals with positive balances. In robustness checks discussed in the results section, we estimate analogous models using both log(balance + 1) and inverse hyperbolic sine transformations of debt balances, and find substantively similar patterns.

We cluster standard errors at the couple or divorce-case level to account for the panel structure of the data. We present our results in terms of the predicted margins of debts held (incidence/balance) at each month since dissolution and interpret differences as statistically meaningful when 95% confidence intervals do not overlap or when formal tests of gender differences are statistically significant. Our goal is to describe within-person debt trajectories around the dissolution process rather than to identify the causal effect of divorce.

## Results

### Sample Characteristics

Table 1 presents descriptive statistics for divorcing mothers and fathers in the analytic sample. Mothers and fathers are, on average, 38 and 40 years old at the time of divorce petition filing. Mothers are slightly more likely than fathers to have earnings in a given month (77% vs. 75%, p < .01), while fathers are more likely to have missing earnings information (19% vs. 16%, p < .01)^6^. Among those with earnings, fathers earn about $1,250 more per month than mothers, on average (p < .01). Child support arrangements also differ sharply by parent gender: about one-quarter of mothers receive child support and one-quarter of fathers pay child support, whereas fewer than 2% of mothers pay or fathers receive child support. Receipt of TANF^7^ and Unemployment Insurance benefits is uncommon, but mothers are more likely than fathers to receive TANF (0.47% vs. 0.04%, p < .01), and fathers are more likely than mothers to receive UI benefits (2.16% vs. 1.36%, p < .01).

Debt holding is common among divorcing parents. Approximately 80% of mothers and 84% of fathers hold some form of debt in a given month. Credit card debt is the most common debt type, followed by auto loans, mortgages, student loans, and personal loans. Mortgage debt accounts for the largest balances, averaging approximately $36,000–$44,000 in a given month when zero balances are included and $88,000–$91,000 among those with positive balances. Personal loans have the lowest average outstanding balances. Fathers are more likely than mothers to hold debt and carry larger balances across most debt categories. Student loans are the exception: mothers are more likely than fathers to hold student debt and carry higher student loan balances. We return to student loans below as a placebo-style outcome because, unlike asset-backed and unsecured debts, they are less likely to be directly reorganized through the divorce process.

### Debt Trajectories over the Anticipatory and Recovery Phases of Divorce

#### Overall Debt

Figure 2 shows predicted debt incidence and balances for mothers and fathers over the dissolution process, estimated from Eq. (1). Overall debt trajectories provide evidence of anticipatory deleveraging before divorce petition filing. There is less evidence, at the aggregate level, of debt procurement or debt transfer. During recovery, debt trajectories are most consistent with a temporary crisis pattern, although mothers’ lower post-petition incidence of debt also suggests possible longer-term strain.

During the anticipatory phase, the predicted probability of holding any debt declines earlier and more sharply for mothers than for fathers. Mothers’ debt incidence begins falling about one year before petition filing, while fathers’ incidence begins declining about four months before petition filing. Fathers are about four percentage points more likely than mothers to hold any debt throughout much of the anticipatory phase, and this gap grows as mothers’ predicted probability of debt holding declines more quickly before petition. Overall balances also decline substantially before petition filing: mothers’ and fathers’ balances fall by more than $10,000 over the anticipatory period. Although mothers and fathers begin the period with similar predicted balances, their trajectories begin to diverge around 12 months before petition filing, as fathers’ balances increase while mothers’ balances continue to decline; however, this difference is not statistically significant.

In the recovery phase, debt incidence stabilizes for both mothers and fathers, but does not return to early anticipatory levels for mothers. By the end of the recovery period, mothers and fathers do not significantly differ in their predicted probability of holding any debt, although fathers remain about five percentage points higher in magnitude. Overall debt balances decline through approximately seven months after petition filing and then trend upward for both mothers and fathers. This pattern suggests that much of the decline in total debt occurs before or shortly after the formal start of the dissolution process, while debt balances begin to rebuild during recovery. By the end of the observation period, mothers and fathers do not significantly differ in overall debt balances.

#### Heterogeneity by Debt Type

The overall results mask important differences across debt types. Consistent with our theoretical expectations, asset-backed debts — mortgages and auto loans — show the clearest evidence of anticipatory deleveraging and post-petition recovery. In contrast, unsecured debts — credit card debt and personal loans — are more stable or decline over the dissolution process, providing less evidence that parents, on average, increase reliance on unsecured credit in response to divorce.

#### Asset-Backed Debt

Mortgage and auto debt follow trajectories consistent with the economic unwinding of jointly held or jointly used household investments. The predicted probability of holding mortgage debt declines during the anticipatory phase and begins to stabilize or trend upward during recovery. At the start of the observation period, fathers are more likely than mothers to hold mortgage debt: predicted mortgage incidence is approximately 25%–34% for mothers and 30%–40% for fathers. This gender gap grows and is statistically significant from approximately 13 months before petition filing through eight months after petition filing, before narrowing and becoming statistically nonsignificant toward the end of recovery. Although mortgage incidence begins to rise later in the recovery period, levels at the end of the observation period are not statistically different from their post-petition minimum.

Auto loans show a similar but less pronounced pattern. Fathers are about five percentage points more likely than mothers to hold auto debt from roughly 13 months before petition filing through six months afterward, after which mothers’ and fathers’ probabilities converge. For both mothers and fathers, the probability of holding auto debt declines by approximately five percentage points during the anticipatory phase and increases modestly during recovery.

Balance trajectories reinforce this pattern. Mortgage and auto balances decline in anticipation of petition filing, consistent with deleveraging, but begin to recover during the post-petition period. Predicted mortgage balances dip by about $8,000 in the five months preceding petition filing and then recover quickly afterward for both mothers and fathers. Fathers’ mortgage balances remain higher than mothers’ by approximately $10,000 through the early recovery phase, with statistically significant differences from about five months before petition filing through nine months after. By the end of recovery, mortgage balances are statistically similar for mothers and fathers and are approximately $10,000–$12,000 higher than early anticipatory levels, although these increases are not statistically significant.

Auto debt balances also decline through the anticipatory period and early recovery, before trending upward later in recovery. Fathers’ predicted auto loan balances are about $1,300 higher than mothers’ throughout much of the observation period, with statistically significant differences from 14 months before petition filing through seven months afterward. The later upward trend in auto balances is not statistically significant, but it is consistent with parents gradually reestablishing access to asset-backed credit as they form separate households.

Taken together, these patterns suggest that asset-backed debts are strongly implicated in the economic uncoupling of divorce. Mortgage and auto debt decline before petition filing, consistent with deleveraging or the reallocation of jointly held obligations, but show signs of recovery after petition filing as parents stabilize their post-divorce financial arrangements.

#### Unsecured Debt

Unsecured debts follow a different pattern. Credit card debt, the most common form of debt in the sample, changes less sharply than asset-backed debt. Fathers are approximately three to five percentage points more likely than mothers to have a credit card balance from six months before petition filing through four months after, but this difference disappears later in recovery. Mothers’ probability of holding credit card debt falls around petition filing and then rises toward pre-petition levels during recovery, while fathers’ credit card incidence remains more stable.

Credit card balances decline over the dissolution process for both mothers and fathers. Fathers hold approximately $1,200 more credit card debt than mothers from one year before petition filing through one year afterward, with statistically significant differences between one year before petition and seven months after. Over time, however, mothers’ and fathers’ balances converge as fathers’ balances fall more steeply than mothers’.

Personal loan debt shows the clearest evidence of sustained decline. The predicted probability of holding personal loan debt is low for both mothers and fathers, and although fathers appear slightly more likely than mothers to hold personal loans from approximately 12 months before petition filing through 13 months afterward, the magnitude of this difference is small. Beginning roughly six months before petition filing, personal loan incidence declines for both parents, with a sharper decline among mothers. Personal loan balances also fall across the dissolution process for both mothers and fathers. Fathers hold approximately $600 more in personal loan debt than mothers throughout much of the period, with statistically significant differences in the five months leading up to petition filing.

Overall, unsecured debt does not appear to increase during recovery in the way we might expect if parents were broadly relying on credit cards or personal loans to cope with the financial shock of divorce. Instead, credit card and personal loan balances remain stable or decline. These patterns are consistent with deleveraging of unsecured debt, potentially because debt-related financial distress precipitated divorce or because parents reduce unsecured obligations as they reorganize their financial lives (Bridges and Disney 2016; Dew 2007, 2008, 2011). At the same time, mothers’ credit card incidence rises during recovery, suggesting that unsecured credit may remain an important margin of adjustment for mothers even as average balances decline.

The main debt results support our expectation that debt trajectories vary by debt type. Asset-backed debts are most responsive to the dissolution process: mortgage and auto debts decline in anticipation of petition filing and show signs of recovery afterward. These patterns are consistent with deleveraging, debt transfer, and the reorganization of household investments during divorce. By contrast, unsecured debts do not show evidence of broad-based debt procurement during recovery. Credit card and personal loan balances generally decline, suggesting that parents are not, on average, increasing high-cost unsecured debt as they move through the dissolution process. However, mothers’ credit card incidence rises during recovery, which may indicate a gendered reliance on unsecured credit at the extensive margin.

These findings align most closely with a temporary crisis pattern for asset-backed debt, particularly mortgage debt, which declines before petition filing and begins to recover afterward. The evidence for unsecured debt is more mixed. Declining balances are consistent with deleveraging, but mothers’ post-petition increase in credit card incidence suggests that unsecured credit may still reflect financial strain for some parents during recovery.

Our findings are consistent with recent work emphasizing heterogeneity in post-divorce recovery rather than a uniform temporary crisis or chronic strain trajectory (Leopold and Kalmijn, 2024). Asset-backed debt balances show the clearest evidence of recovery after petition filing, while unsecured debt and mothers’ credit card incidence point to more uneven adjustment. Thus, debt trajectories after divorce appear to depend not only on parent gender but also on the type of liability being reorganized.

#### Student Loans

Student loans provide a useful placebo-style comparison because they are typically individually held, often acquired before marriage, and less likely than the other debts we study to be directly reorganized through the divorce process. If our main results are capturing debt changes associated with economic uncoupling rather than general artifacts of the data or model, we would expect student loan trajectories to show less evidence of inflection around petition filing.

This is what we find. Mothers are more likely than fathers to hold student loans and have higher student loan balances at the beginning of the observation period. However, student loan incidence and balances decline steadily over time for both mothers and fathers, without a perceptible inflection around petition filing. Mothers’ student loan balances decline more sharply than fathers’, and gender differences narrow by the end of the observation period, but these changes appear gradual rather than tied to the dissolution process. The absence of a clear petition-related shift in student loans increases confidence that the patterns observed for asset-backed and unsecured debts reflect debt-specific responses to divorce rather than general trends in all credit accounts.

### Testing Mechanisms: Financial Strain and Asset-Backed Debt Ownership

To what extent do these trajectories reflect financial strain during the dissolution process? To examine this possibility, we estimate supplementary models predicting repayment struggles using an indicator for delinquency. Stable or falling delinquency would suggest that declining unsecured balances do not simply reflect worsening repayment difficulty, whereas rising delinquency would indicate increasing debt-related financial distress.

**Figure 5:**
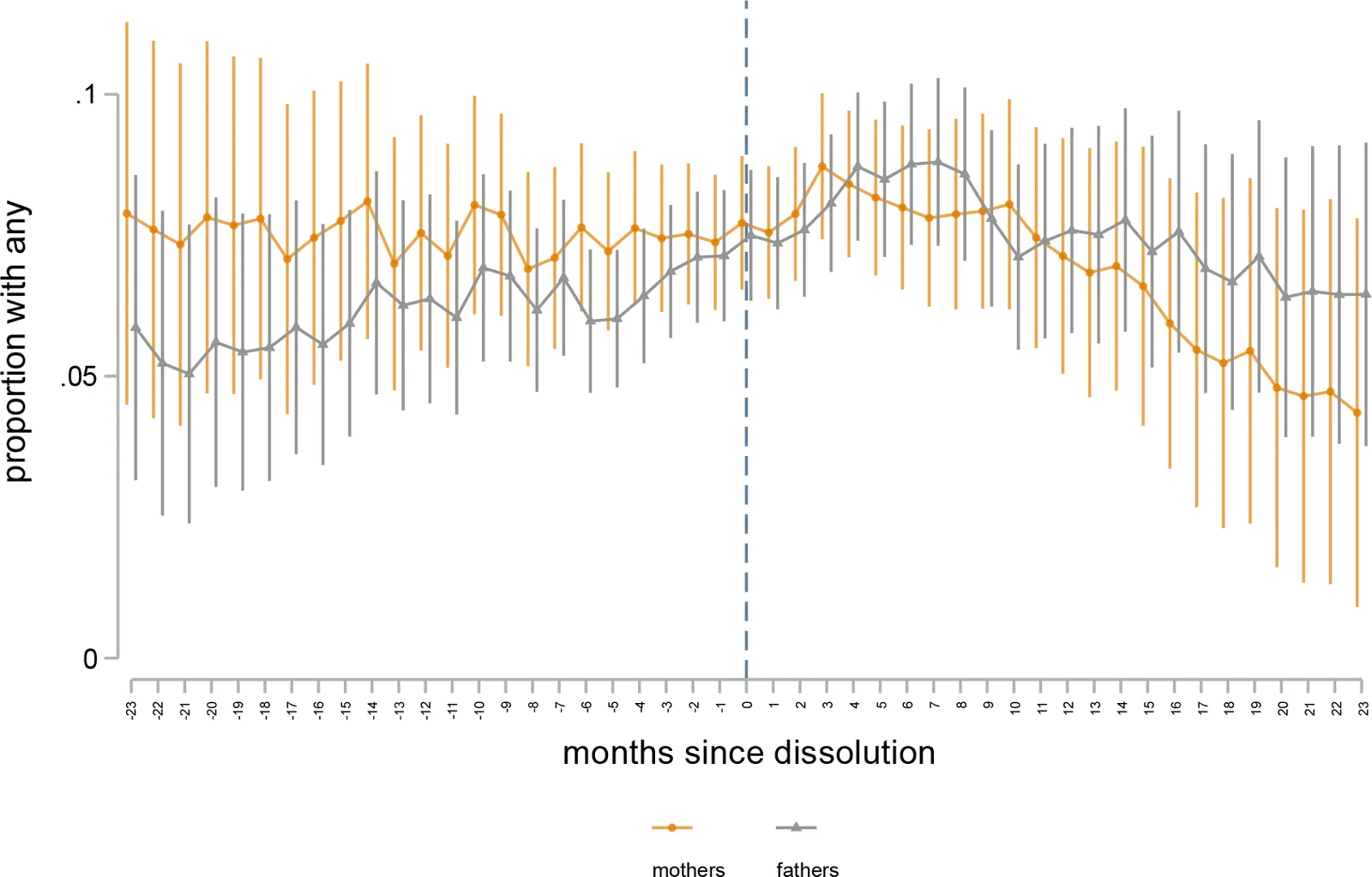
Predicted delinquency rates over months since dissolution, by parent **Note:** Figure plots predicted values of the rate of delinquency in any debt for those with a positive overall debt balance at each month since dissolution. Models control for monthly wages, any positive wages, parent’s age, and indicators for child support receipts and payments, TANF and UI benefits receipts. Error bars show 95% confidence intervals. Errors are clustered at the divorce case level. Estimates are weighted to adjust for different sampling rates across counties. **Source:** The OSU-UWCP, WADC, and Wisconsin CRD

Figure 5 shows predicted delinquency rates for mothers and fathers. At the beginning of the observation period, approximately 6% of mothers and fathers have a delinquent account. Delinquency rises during the anticipatory phase and peaks at approximately 9% around four months after petition filing. It then declines steadily, reaching just over 4% for mothers and about 5% for fathers by the end of the recovery period. Mothers and fathers have similar delinquency levels and trends throughout the dissolution process.

These patterns suggest that debt-related financial distress rises before and shortly after petition filing but declines during recovery. This trajectory is consistent with the temporary crisis model: delinquency increases during the period of greatest disruption but improves as parents move into more stable post-petition arrangements. The decline in delinquency also helps interpret the unsecured debt findings. Because credit card and personal loan balances decline rather than rise during recovery, and delinquency falls after its early post-petition peak, the evidence does not suggest a broad shift toward worsening unsecured debt burden in the average case.

A second mechanism concerns the ownership and liquidation of asset-backed debt. The decline in unsecured balances and delinquency may partly reflect deleveraging of asset-backed debt, especially mortgages. If parents sell homes, refinance mortgages, or otherwise reduce mortgage obligations during the dissolution process, this may generate liquidity that allows them to pay down other debts. To explore this mechanism, we examine trajectories of auto, credit card, and personal loan balances separately for individuals whose mortgage balances always remain positive, those whose mortgage balances fall from positive to zero at some point during the dissolution process, and those who never have mortgage debt.

**Figure 6:**
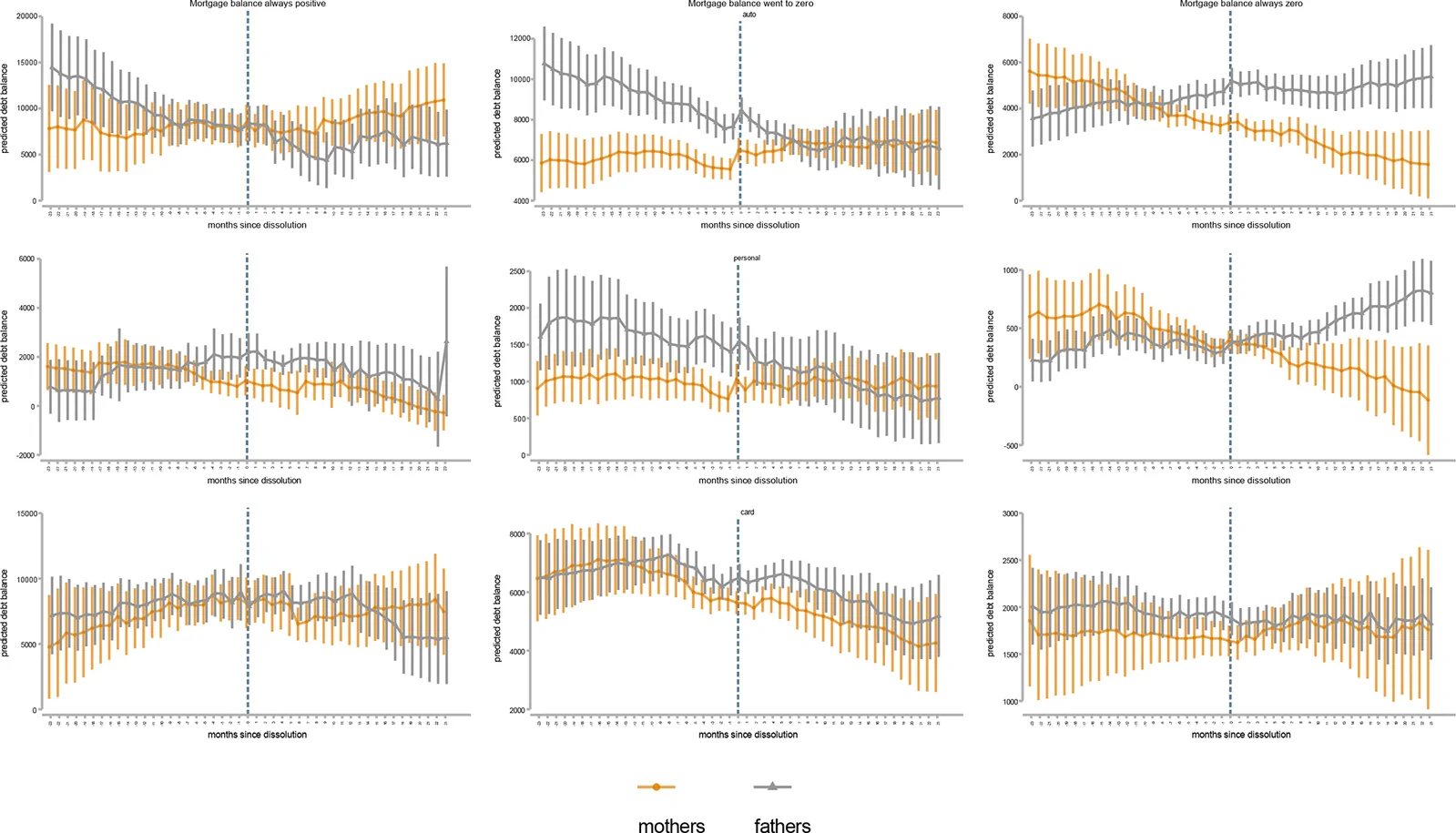
Predicted balance in auto, personal loan, and credit card debt over months since dissolution, by mortgage deleveraging scenarios **Notes:** Figure plots predicted balance (in dollars) of auto debt at each month since dissolution. Y-axis ranges differ across graphs to account for differing magnitudes across debt types. Models control for monthly wages, any positive wages, parent’s age, and indicators for child support receipts and payments, TANF and UI benefits receipts. Error bars show 95% confidence intervals. Errors are clustered at the divorce case level. Means are weighted to adjust for different sampling rates across counties. **Source:** OSU-UWCP, the Wisconsin Administrative Data Core, and the Wisconsin Court Records Data.

Figure 6 shows that changes in non-mortgage debt are concentrated among parents whose mortgage balances fall from positive to zero^8^. Among those with continuously positive mortgage balances, mothers’ non-mortgage balances rebound more quickly after petition filing, while fathers’ balances decline, although gender differences are not statistically significant. Among those who never have mortgage debt, debt levels are lower overall, and trajectories differ from the full-sample pattern: mothers’ auto and personal loan balances decline throughout the dissolution process, while fathers’ balances increase, and credit card balances remain closer to pre-petition levels. These subgroup patterns suggest that mortgage deleveraging may play an important role in the broader debt trajectories observed in the main analyses. In particular, selling or otherwise exiting mortgage debt may allow some parents to reduce other obligations during divorce.

### Robustness Checks and Sensitivity Analyses

#### Debt Allocation within Couples

The allocation of debt within marriage is difficult to observe directly. Wisconsin is a community property state, meaning that debts acquired during marriage are generally presumed to be shared marital obligations. In practice, however, couples may manage repayment in different ways: one spouse may be primarily responsible for a debt, spouses may share repayment equally or unequally, or a debt may appear on both spouses’ credit reports even if repayment responsibility is negotiated informally. Because credit report data identify balances reported on each individual’s credit file but do not directly observe legal or practical responsibility for repayment, our main analyses attribute balances to the individual on whose credit report they appear, except when both spouses have exactly equal balances in the same debt category and month, in which case we assign half the balance to each spouse.

To assess sensitivity to this measurement assumption, we estimate two alternative specifications. First, under a *partial separation of debt* scenario, we treat some debts appearing on only one spouse’s credit report as potentially shared. When one spouse has a positive balance and the other has a zero balance for a given debt type in the pre-dissolution period, we assign half of the positive balance to each spouse. We continue to divide exactly equal balances in half, as in the main specification. Second, under a *no separation of debt* scenario, we assume all debt is shared within the couple: we sum spouses’ balances and assign half of the total to each spouse. Appendix Table A1 reports the number of observations affected by these assumptions across debt categories. Mortgage debt is most likely to appear jointly held under our exact-match definition, while other debts are more often held in one spouse’s name. Cases in which both spouses hold positive but unequal balances are relatively rare.

**Figure 7:**
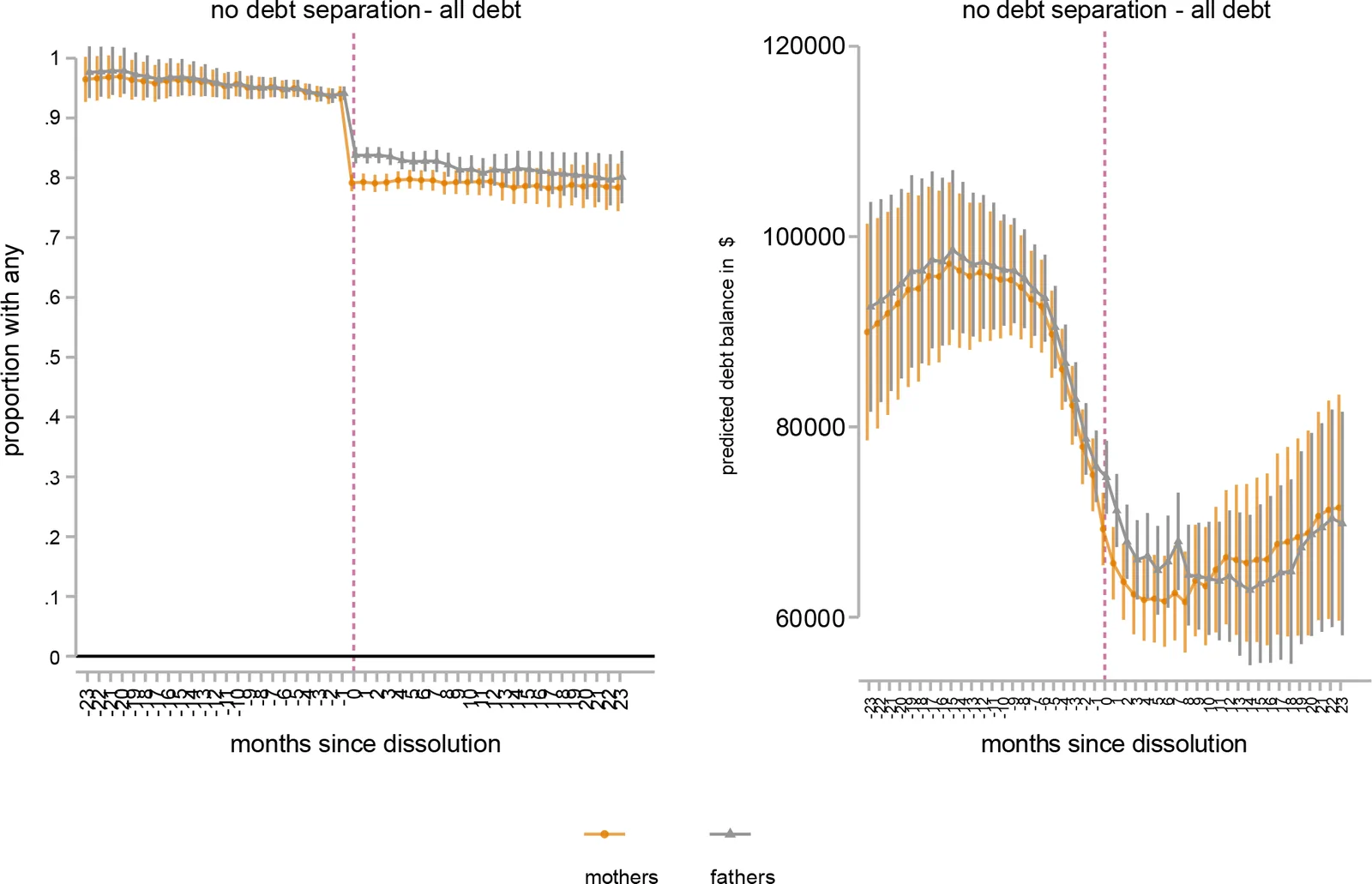
Incidence and balance of overall debt in no debt separation scenario **Notes:** Figure plots predicted incidence and balance (in dollars) of overall debt at each month since dissolution. Models control for monthly wages, any positive wages, parent’s age, and indicators for child support receipts and payments, TANF and UI benefits receipts. Error bars show 95% confidence intervals. Errors are clustered at the divorce case level. Means are weighted to adjust for different sampling rates across counties. **Source:** The OSU-UWCP, the Wisconsin Administrative Data Core, and the Wisconsin Court Records Data.

Figure 7 shows trajectories of overall debt incidence and balances under the no separation scenario. Results for the partial separation scenario are shown in Appendix Figure A6 and are similar. Under the no separation scenario, gender differences in overall debt incidence and balances during the anticipatory phase largely disappear, as expected when debt is mechanically assigned equally within couples. The change in debt incidence from the anticipatory to recovery phase is larger under this scenario than in the main specification for both mothers and fathers. Predicted balances decline by approximately $30,000 from the anticipatory to recovery phase for both parents. These results suggest that our main specification provides a conservative estimate of changes in debt incidence and yields broadly similar conclusions about pre- to post-petition changes in debt balances.

### Alternative Balance Specifications

Debt balances are right-skewed and include a nontrivial number of zero balances. Our preferred specification models balances in levels, including zeros, to preserve the interpretation of debt as dollar-denominated exposure in the full sample rather than conditioning on individuals who hold positive balances. To assess sensitivity to skewness, we estimate analogous models using both log(balance + 1) and inverse hyperbolic sine transformations of debt balances. Results from these alternative specifications, discussed in Appendix Section A.1.1 and shown in Appendix Figures A7–A8, are substantively similar to those from our preferred specification.

## Discussion and Conclusion

This study extends research on the economic consequences of divorce by examining debt as a dynamic component of post-divorce economic well-being. Prior work has focused primarily on income, employment, assets, and wealth, leaving less known about how liabilities change as couples financially separate. Yet debt is central to household financial portfolios and is closely linked to relationship formation, stability, and quality. Using linked Wisconsin court records, monthly credit report data, and administrative records of income and benefit receipt, we traced debt trajectories among divorcing parents before and after divorce petition filing. We examined whether these trajectories differed by parent gender and debt type, distinguishing between asset-backed debts, such as mortgages and auto loans, and unsecured debts, such as credit cards and personal loans.

We find that debt holdings generally decline during the dissolution process for both mothers and fathers, but the timing and magnitude of these changes vary substantially by debt type and parent gender. Asset-backed debts show the clearest evidence of anticipatory deleveraging and post-petition recovery. Mortgage debt, in particular, declines before petition filing and then begins to recover during the recovery period, a pattern consistent with prior evidence that divorce is associated with changes in housing wealth and homeownership (Kapelle & Baxter, 2020; Leopold, 2018). Auto debt follows a similar, though less pronounced, trajectory. These findings suggest that asset-backed debt is strongly implicated in the economic uncoupling of divorce, likely because homes and vehicles are central household investments that must be retained, sold, refinanced, or otherwise reorganized as parents establish separate households.

Unsecured debts follow a different pattern. Rather than increasing sharply after divorce petition filing, credit card and personal loan balances generally decline over the dissolution process. This provides little evidence that divorcing parents, on average, broadly increase reliance on unsecured credit to manage the financial shock of divorce. Instead, the findings are more consistent with deleveraging of unsecured obligations, potentially because debt-related financial strain helped precipitate divorce or because parents reduce constraining debts as they reorganize their financial lives (Bridges & Disney, 2016; Dew, 2007, 2008, 2011). At the same time, mothers’ credit card incidence rises during recovery, suggesting that unsecured credit may remain an important margin of adjustment for some mothers even as average balances decline.

Taken together, the results provide more support for a temporary crisis pattern than for a chronic strain pattern with respect to asset-backed debt balances. Mortgage and auto debt decline in anticipation of petition filing but begin to recover afterward, suggesting that some forms of credit may help parents reestablish housing, transportation, and household stability after divorce. The evidence for unsecured debt is more mixed. Declining unsecured balances are consistent with deleveraging, but mothers’ post-petition increase in credit card incidence suggests that recovery may be uneven across debt types and across margins of debt holding. The convergence in mothers’ and fathers’ debt holdings suggests that divorce narrows some gender differences in credit portfolios, although this convergence should not be interpreted straightforwardly as improved economic well-being. Convergence can occur because fathers reduce debt, because mothers lose access to some forms of credit, because mothers take on new unsecured debt, or because both parents experience shifts in household assets and obligations. In other words, becoming more similar in debt holdings does not necessarily mean that mothers become more economically secure; it may instead reflect a reorganization of financial constraint, credit access, and repayment burdens after divorce.

The mechanism analyses provide further insight into these patterns. Delinquency rises during the anticipatory phase and peaks shortly after petition filing, but then declines during recovery for both mothers and fathers. This pattern suggests that debt-related financial distress increases during the most disruptive period of the divorce process but improves as parents move into more stable post-petition arrangements. The mortgage subgroup analyses are also consistent with the possibility that exiting asset-backed debt, particularly mortgage debt, creates liquidity that can be used to reduce other obligations. Parents whose mortgage balances fall from positive to zero account for much of the decline in auto, credit card, and personal loan balances. This interpretation aligns with prior research showing that wealth can buffer families against disruptive life events (Rhodes et al., 2024; Rodems & Pfeffer, 2021). However, our data do not directly observe home sales, home equity, refinancing, or the use of proceeds. We therefore interpret these results as suggestive evidence that asset-backed deleveraging may help reduce other debts, rather than direct evidence that mortgage equity is used to repay unsecured obligations.

The placebo-style student loan results strengthen this interpretation. Unlike asset-backed and unsecured debts, student loans decline gradually over time and show no clear inflection around petition filing. This pattern is consistent with expectations for a debt type that is typically individually held, often acquired before marriage, and less likely to be reorganized through the divorce process. The absence of a petition-related shift in student loans increases confidence that the observed changes in mortgage, auto, credit card, and personal loan debt are not simply artifacts of aging, repayment schedules, or the model structure, but instead reflect debt-specific responses to the dissolution process.

Our study has several limitations. First, we do not directly observe how couples share responsibility for debts within marriage. Credit report data identify balances reported on each individual’s credit file, but they do not reveal whether debts were jointly incurred, how repayment responsibilities were negotiated, or whether balances appearing on both spouses’ reports represent the same liability. We address this limitation by estimating sensitivity checks under alternative assumptions about debt sharing, showing that the magnitude of change varies across assumptions but that the broad patterns remain similar. Second, because we observe credit liabilities but not assets or total net worth, we cannot determine whether changes in debt represent worsening financial position, improved balance sheets, asset liquidation, or changes in access to credit. his is especially important because debt and assets are often jointly embedded in household wealth portfolios, particularly through housing (Killewald, Lee, and England 2023). We therefore interpret debt trajectories as changes in liabilities and credit exposure, not as direct measures of wealth or economic well-being. Third, while our fixed-effects design captures within-person changes over the dissolution process, the estimates remain descriptive rather than causal. Time-varying unobserved factors, including financial knowledge, health, disability, family support, or changes in employment circumstances, may shape both divorce trajectories and debt holdings. Fourth, we do not observe repartnering or remarriage, which may affect post-divorce household composition, financial resources, and credit access. The relatively short post-petition window may limit the extent to which repartnering affects observed trajectories^9^, but future work with longer follow-up should examine this directly. Fifth, although mortgage trajectories are central to our interpretation, we observe mortgage balances rather than homeownership, home values, home sales, or equity extraction. We therefore treat mortgage exit and changes in other debt balances as suggestive of asset-backed deleveraging, not direct evidence of how housing wealth is used during divorce. Finally, our results are situated in Wisconsin, a community property state, and debt trajectories may differ in states with different laws governing marital property, liability, and divorce.

Despite these limitations, this study sheds light on an understudied dimension of the dynamic economic uncoupling of divorce. Divorce involves not only changes in income, household composition, and asset ownership, but also the allocation, reduction, and rebuilding of liabilities. Our findings show that debt is not a uniform marker of economic strain. Asset-backed and unsecured debts follow distinct trajectories, suggesting that some debts may operate as resources in the process of household reorganization, while others may reflect financial strain or deleveraging. Access to asset-backed debt, particularly mortgage debt, may shape whether divorce-related financial disruption resembles a temporary crisis or more persistent strain.

These findings point to several directions for future research. First, future work should examine how couples share, negotiate, and perceive liabilities during marriage, and how these arrangements shape the division of debt during divorce. Second, comparative research across states could assess how marital property regimes and debt liability rules influence debt reallocation and post-divorce financial outcomes. Third, studies with longer follow-up periods should examine how post-divorce household composition, children’s residence, child support, repartnering, and remarriage affect debt recovery. Finally, future research should investigate new credit account openings after divorce to better distinguish debt procurement from debt transfer and deleveraging. Doing so would clarify whether new debt represents financial strain, restored access to credit, or investment in post-divorce household stability.

## Supplementary Material

Supplementary Files

This is a list of supplementary files associated with this preprint. Click to download.

• Appendix.docx

## Figures and Tables

**Figure 1:**
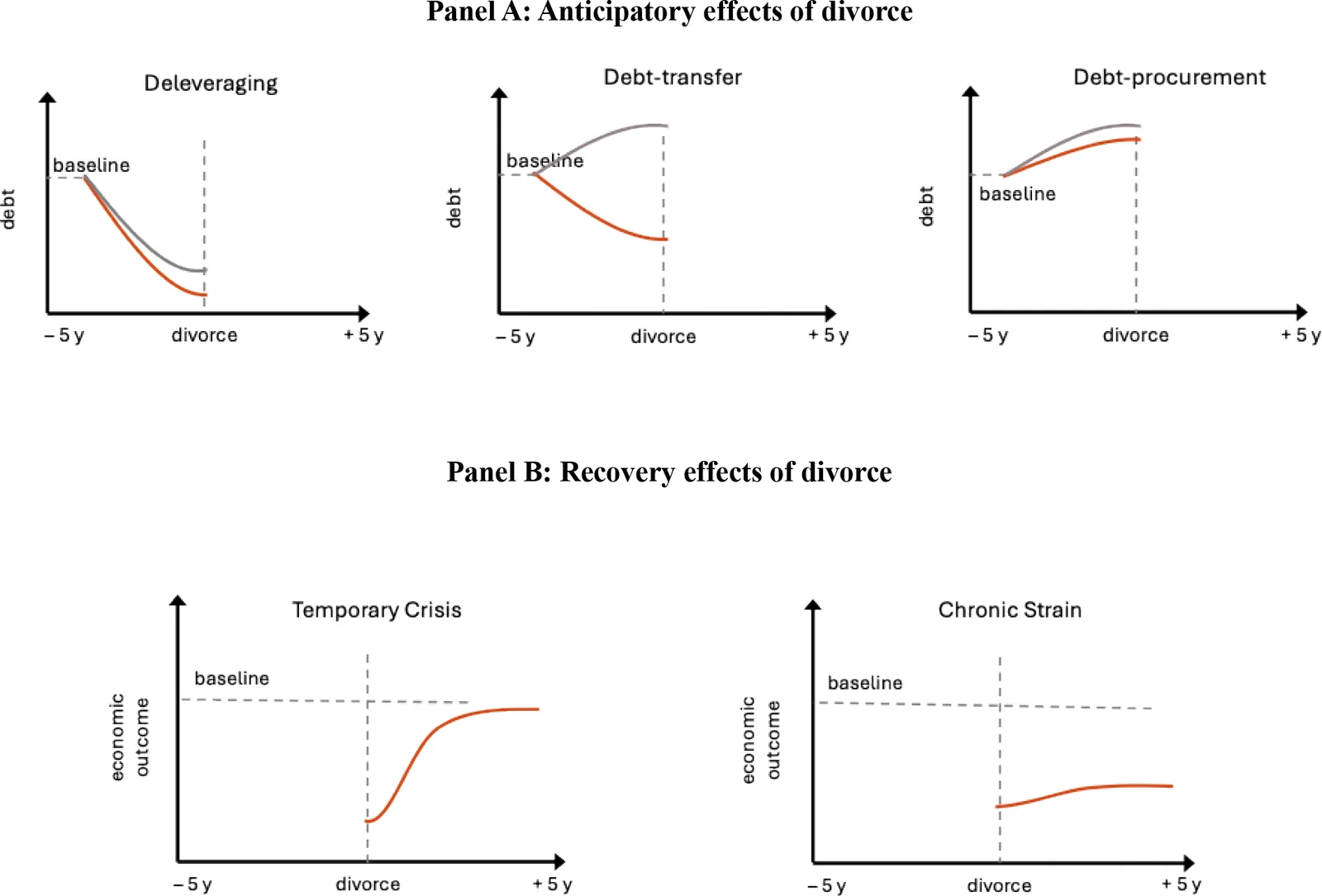
A theoretical framework for divorce as a dynamic decoupling of debt **Notes:** Figure shows theoretical predictions about the anticipatory (Panel A) and recovery (Panel B) effects of divorce on debt holdings.

**Figure 2:**
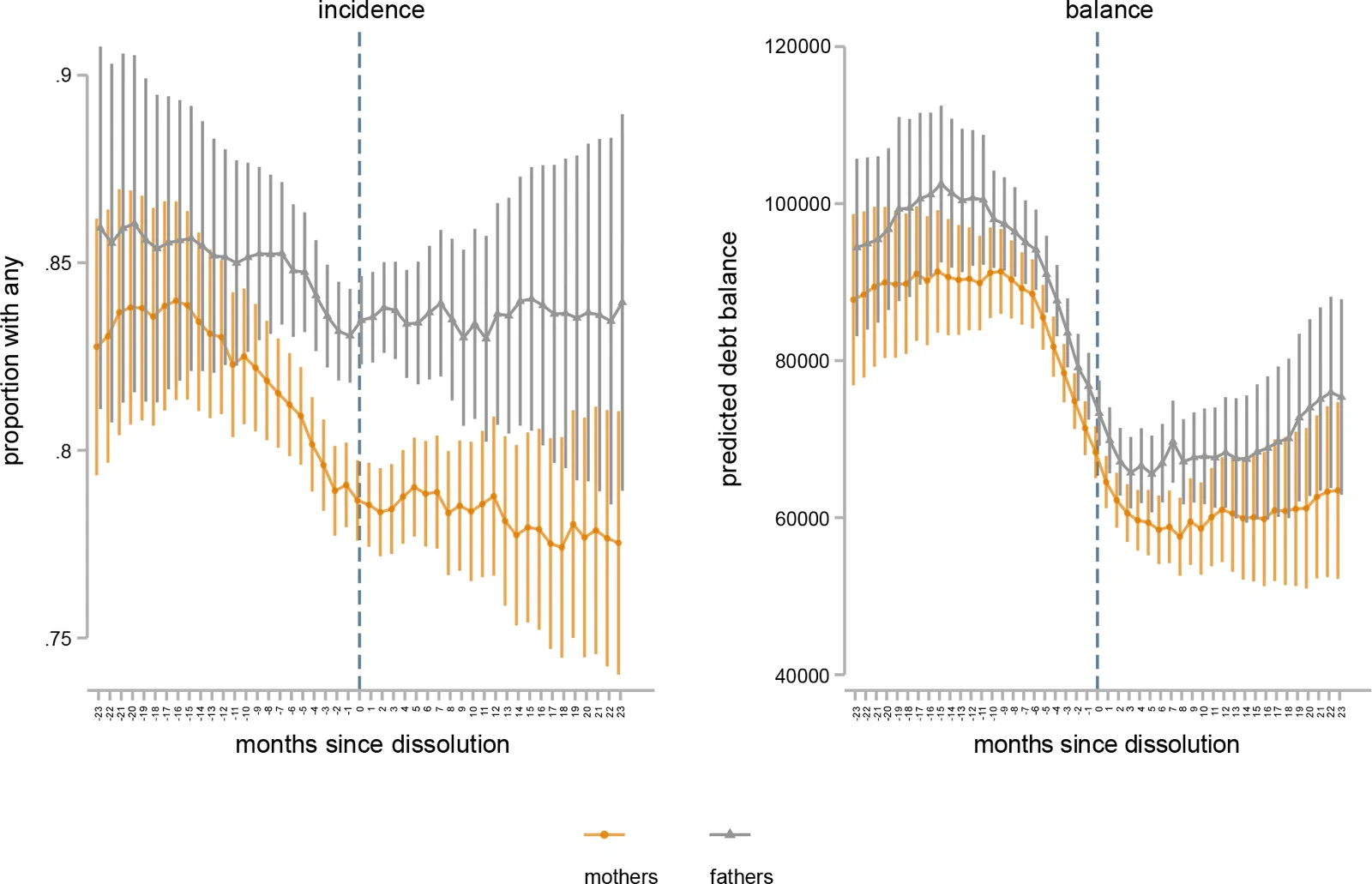
Incidence and balance of overall debt, by parent **Note:** Figure plots predicted incidence (left) and balance in dollars (right) of all debt at each month since dissolution. Models control for monthly wages, any positive wages, parent’s age, and indicators for child support receipts and payments, TANF and UI benefits receipts. Error bars show 95% confidence intervals. Errors are clustered at the unit level. Estimates are weighted to adjust for different sampling rates across counties. N = 1,834 mothers and 1,834 fathers. **Source:** The OSU-UWCP, WADC, and Wisconsin CRD.

**Figure 1:**
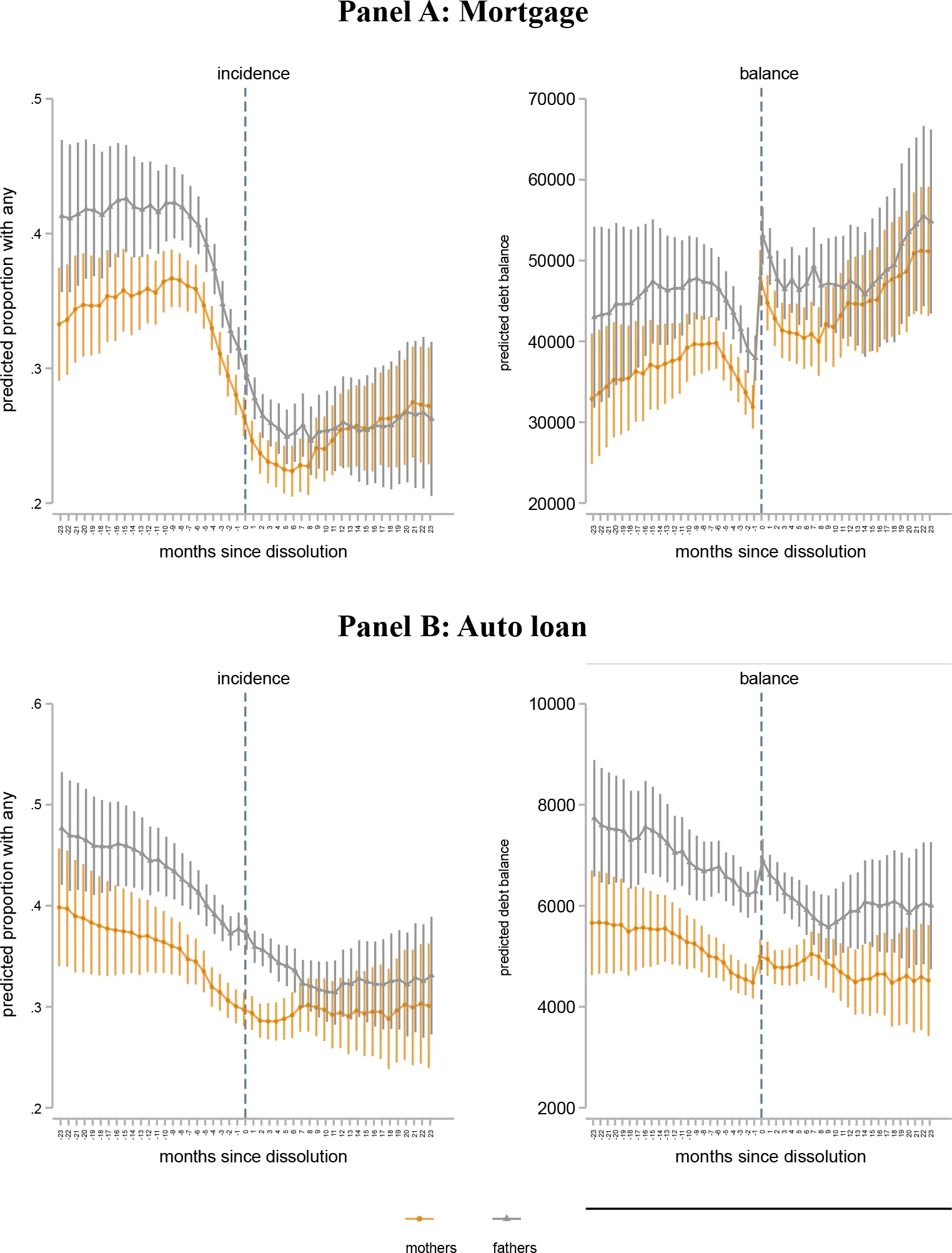
Predicted incidence and balance of asset-backed debt over months since dissolution, by parent **Note** Figure plots predicted incidence (left) and balance in dollars (right) of asset-backed (mortgage-Panel A and auto loan-Panel B) debt at each month since dissolution. Models control for monthly wages, any positive wages, parent’s age, and indicators for child support receipts and payments, TANF and UI benefits receipts. Error bars show 95% confidence intervals. Errors are clustered at the divorce case level. Estimates are weighted to adjust for different sampling rates across counties. N = 1,834 mothers and 1,834 fathers. **Source:** The OSU-UWCP, WADC, and Wisconsin CRD.

**Figure 2:**
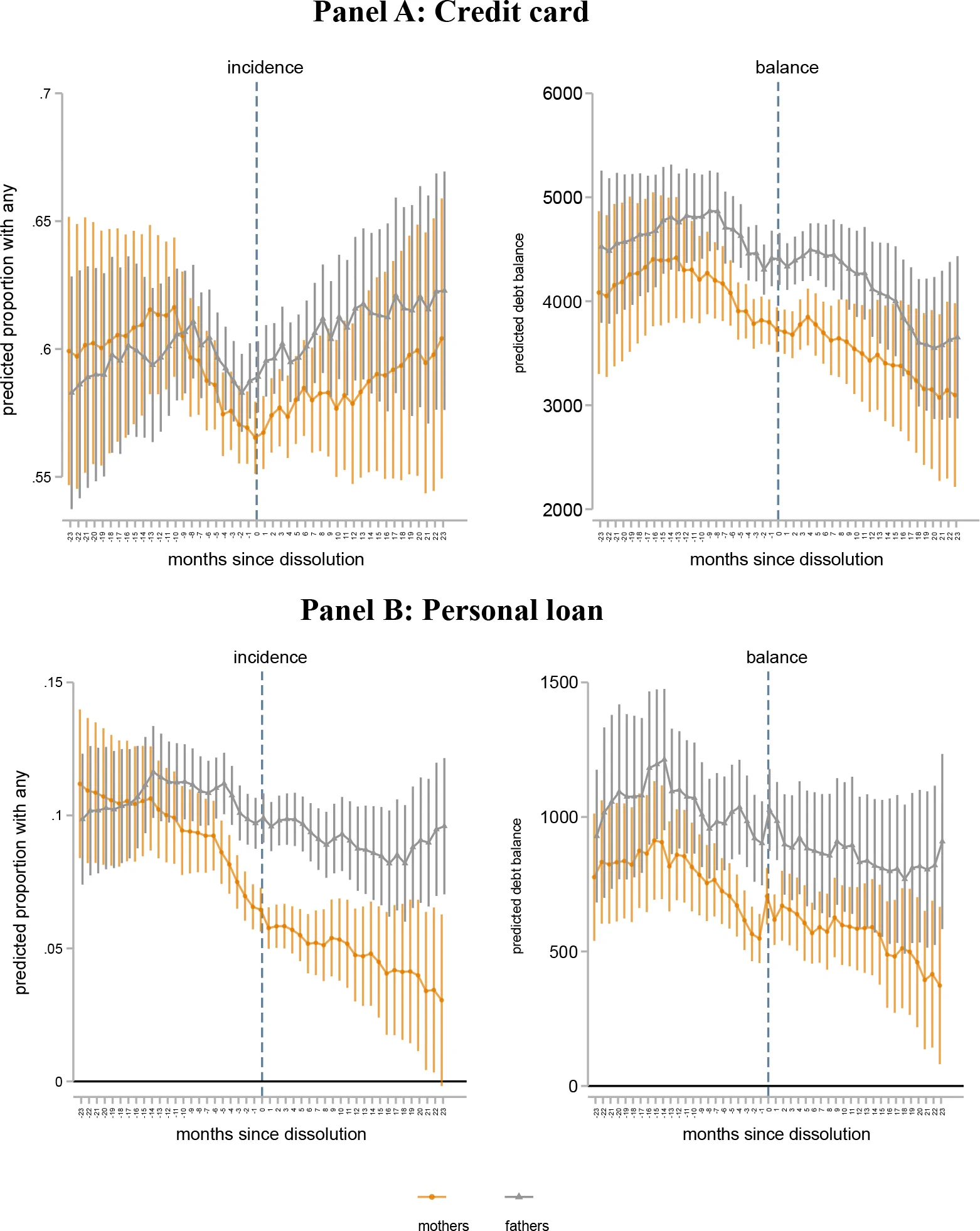
Predicted incidence and balance of unsecured debt over months since dissolution, by parent **Note:** Figure plots predicted incidence (left) and balance in dollars (right) of unsecured (credit card-Panel A and personal loan-Panel B) debt at each month since dissolution. Models control for monthly wages, any positive wages, parent’s age, and indicators for child support receipts and payments, TANF and UI benefits receipts. Error bars show 95% confidence intervals. Errors are clustered at the divorce case level. Estimates are weighted to adjust for different sampling rates across counties. N = 1,834 mothers and 1,834 fathers. **Source:** The OSU-UWCP, WADC, and Wisconsin CRD.

**Figure 3:**
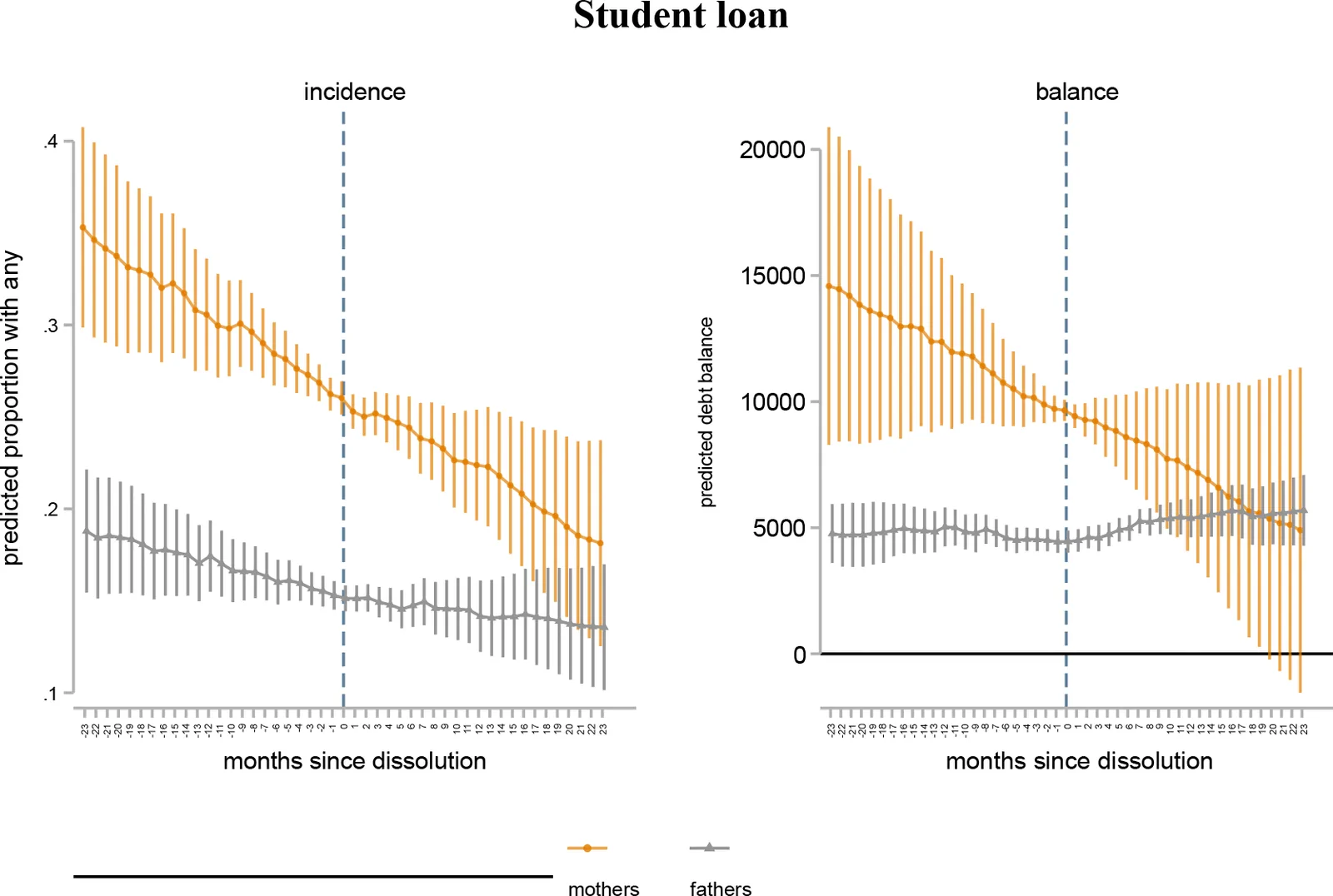
Predicted incidence and balance of student loan debt over months since dissolution, by parent **Note:** Figure plots predicted incidence (left) and balance in dollars (right) of student loan debt at each month since dissolution. Models control for monthly wages, any positive wages, parent’s age, and indicators for child support receipts and payments, TANF and UI benefits receipts. Error bars show 95% confidence intervals. Errors are clustered at the divorce case level. Estimates are weighted to adjust for different sampling rates across counties. N = 1,834 mothers and 1,834 fathers. **Source:** The OSU-UWCP, WADC, and Wisconsin CRD.

**Table 1:** Sample characteristics

*Panel A: Individual Characteristics*			

	Mothers	Fathers	*t*-test
	
Observations (persons)	1,834	1,834	
Observations (person-months)	86,198	86,198	
Age	38.07 (7.36)	40.29 (8.02)	***
% with any wages in a month	77.34% (41.87)	75.21% (43.54)	***
% with missing wages in a month	16.35% (36.98)	19.00% (39.23)	***
Monthly wages when positive	3,647.36 (3,183.41)	5,412.16 (4,988.89)	***
Monthly child support received	25.13% (43.38)	1.52% (12.23)	***
Monthly child support paid	1.36% (11.59)	24.44% (42.97)	***
Monthly TANF receipts	0.47% (06.82)	0.04% (02.04)	***
Monthly UI benefits receipts	1.36% (11.57)	2.16% (14.54)	***
All debt			
Incidence	79.51% (40.36)	84.23% (36.44)	**
Balance	70,102.85 (110,000.00)	76,545.14 (120,000.00)	***
Balance when positive	88,167.34 (110,000)	90,874.55 (130,000)	**
Mortgage			
Incidence	28.61% (45.19)	32.93% (47.00)	***
Balance	36,762.88 (78,041.87)	43,710.75 (84,411.03)	***
Balance when positive	128,513.55 (97,000)	132,719.70 (99,000)	***
Auto			
Incidence	33.07% (47.05)	38.94% (48.76)	**
Balance	5,075.00 (9,713.18)	6,715.65 (12,932.87)	***
Balance when positive	15,347.13 (11,000)	17,245.88 (16,000)	***
Student			
Incidence	25.61% (43.65)	14.75% (35.46)	***
Balance	8,924.98 (23,590.53)	4,447.61 (18,490.28)	***
Balance when positive	34,848.48 (36,000)	30,160.92 (39,000)	***
Credit card			
Incidence	56.75% (49.54)	59.17% (49.15)	***
Balance	3,559.13 (7,158.19)	4,097.60 (8,157.26)	***
Balance when positive	6,271.25 (8,600)	6,925.18 (9,600)	***
Personal finance			
Incidence	7.54% (2.64)	10.78% (31.02)	***
Balance	756.37 (4,955.59)	1,100.52 (6,760.60)	***
Balance when positive	10,033.45 (15,000)	10,205.79 (18,000)	

**Note:** Mean (and standard deviations) and percentages presented for individual-month level observations. Sample characteristics are weighted to adjust for different sampling rates across counties.

† *p* < 0.10

* *p* < 0.05

** *p*< 0.01

*** *p*<0.001.

**Source:** The OSU-UWCP, WADC and Wisconsin CRD.

## Footnotes

1 Some couples may have shared credit cards where one spouse is the account owner and the other is an “authorized” user. In such cases, the owner of the account is legally responsible for the debt.

2 We were unable to link individuals who did not have a credit report and those with discrepancies in identifiers between the two datasets.

3 Includes personal installment loans taken from banking institutions.

4 Logistic regressions for debt incidence yield similar results (results available upon request).

5 The Wisconsin CRD includes the date of divorce petition as well as the date of final divorce judgment. We find that changes in debt holdings start long before the divorce judgment, and even before petition filing, and that, by the time of the divorce judgment, have already taken place (see Appendix Figure A-1). These results suggest that date of petition is a more accurate proxy for union dissolution than date of final judgment, and we therefore use the petition date rather than divorce judgment date to approximate the date of marital dissolution in all analyses.

6 Fathers are disproportionately likely to move out of state and have their earnings information in the Wisconsin Unemployment Insurance database discontinued.

7 TANF is a federal assistance program that provides cash aid to low-income families with children, designed especially for households headed by single mothers.

8 Results for overall debt (which is heavily influenced by mortgages) and student loan balances (which do not associate with divorce) are shown in Appendix figures A7 and A8 and follow a similar pattern.

9 Although re-marriage or re-partnership remains an important coping mechanism for recovering from the economic and psychological shocks of divorce (Leopold & Kalmijn, 2024; Mortelmans, 2020), re-marriage rates have been declining in recent years, possibly due to a decline in marriage overall (Raley & Sweeney, 2020). While recent estimates are unavailable, older studies have shown that individuals usually take 3–4 years after the dissolution of their previous marriage to remarry, on average (Kreider & Ellis, 2011). Moreover, remarried women are more likely to maintain individual versus joint savings accounts, and sharing debt is less common within cohabiting relationships (which are likely to form sooner after divorce than marital relationships) than within marriages (Kan & Laurie, 2014). Existing research therefore suggests that re-marriage or re-partnership is unlikely to substantively affect post-separation debt balances of divorcing individuals within the short-term (<2 years) after divorce.

## References

[R1] Addo F. R. (2014). Debt, cohabitation, and marriage in young adulthood. Demography, 51(5), 1677–1701. 10.1007/s13524-014-0333-6 25267281 PMC6045913

[R2] Addo F. R., Houle J. N., & Sassler S. (2019). The Changing Nature of the Association Between Student Loan Debt and Marital Behavior in Young Adulthood. Journal of Family and Economic Issues, 40(1), 86–101. 10.1007/s10834-018-9591-6

[R3] Addo F. R., & Zhang X. (2020). Debt Concordance and Relationship Quality: A Couple-Level Analysis. Journal of Family and Economic Issues, 41(3), 405–423. 10.1007/s10834-020-09687-8 32831532 PMC7440213

[R4] Andreß H. J., Borgloh B., Bröckel M., Giesselmann M., & Hummelsheim D. (2006). The Economic Consequences of Partnership Dissolution—A Comparative Analysis of Panel Studies from Belgium, Germany, Great Britain, Italy, and Sweden. European Sociological Review, 22(5), 533–560. 10.1093/ESR/JCL012

[R5] Bandelj N., Lanuza Y. R., & Ji Z. (2024). Raising Children, Rising Debt: Mortgage Debt Among American Families. Social Sciences, 13(11), 1–21. 10.3390/socsci13110600

[R6] Bartfeld J. (2000). Child support and the postdivorce economic well-being of mothers, fathers, and children. Demography, 37(2), 203–213. 10.2307/2648122 10836178

[R7] Bartfeld J., Ahn H.-M., & Ryu J. H. (2012). Economic Well-Being of Divorced Mothers with Varying Child Placement Arrangements in Wisconsin: Contributions of Child Support and Other Income Sources. Institute for Research on Poverty. University of Wisconsin-Madison.

[R8] Bayaz-Ozturk G., Burkhauser R. V., Couch K. A., & Hauser R. (2018). The Effects of Union Dissolution on the Economic Resources of Men and Women: A Comparative Analysis of Germany and the United States, 1985–2013. Annals of the American Academy of Political and Social Science, 680(1), 235–258. 10.1177/0002716218793608

[R9] Beblo M., & Beninger D. (2017). Do husbands and wives pool their incomes? A couple experiment. Review of Economics of the Household, 15(3), 779–805. 10.1007/s11150-016-9342-0

[R10] Berger L. M., & Houle J. N. (2019). Rising Household Debt and Children’s Socioemotional Well-being Trajectories. Demography, 56(4), 1273–1301. 10.1007/s13524-019-00800-7 31292913

[R11] Bianchi S. M., Subaiya L., & Kahn J. R. (1999). The gender gap in the economic well-being of nonresident fathers and custodial mothers. Demography, 36(2), 195–203. https://doi.org/10.2307/2648108 10332611

[R12] Boertien D., & Lersch P. M. (2021). Gender and Changes in Household Wealth after the Dissolution of Marriage and Cohabitation in Germany. Journal of Marriage and Family, 83(1), 228–242. 10.1111/jomf.12705

[R13] Bonnet C., Garbinti B., & Solaz A. (2021). The flip side of marital specialization: The gendered effect of divorce on living standards and labor supply. Journal of Population Economics, 34(2), 515–573. 10.1007/s00148-020-00786-2

[R14] Brevoort K. P., & Kambara M. (2017). CFPB Data Point: Becoming Credit Visible. The CFPB Office of Research. https://files.consumerfinance.gov/f/documents/BecomingCreditVisible_Data_Point_Final.pdf

[R15] Bridges S., & Disney R. (2016). Household Finances, Income Shocks, and Family Separation in Britain. Economic Inquiry, 54(1), 698–718. 10.1111/ecin.12259

[R16] Bryant W. K., & Zick C. D. (2006). The Economic Organization of the Household. Cambridge University Press.

[R17] Callegari J., Liedgren P., & Kullberg C. (2020). Gendered debt – a scoping study review of research on debt acquisition and management in single and couple households. European Journal of Social Work, 23(5), 742–754. 10.1080/13691457.2019.1567467

[R18] Chanda T. (2022). Economic Wellbeing and Labor Supply Patterns of Subsequently Divorcing Mothers in Wisconsin. Journal of Family and Economic Issues, 44, 821–835. 10.1007/s10834-022-09875-8

[R19] De Vaus D., Gray M., Qu L., & Stanton D. (2017). The economic consequences of divorce in six OECD countries. Australian Journal of Social Issues, 52(2), 180–199. 10.1002/ajs4.13

[R20] Dew J. (2007). Two Sides of the Same Coin? The Differing Roles of Assets and Consumer Debt in Marriage. Journal of Family and Economic Issues, 28(1), 89–104. 10.1007/s10834-006-9051-6

[R21] Dew J. (2008). Debt Change and Marital Satisfaction Change in Recently Married Couples. Family Relations, 57(1), 60–71. 10.1111/j.1741-3729.2007.00483.x

[R22] Dew J. (2011). The Association Between Consumer Debt and the Likelihood of Divorce. Journal of Family and Economic Issues, 32(4), 554–565. 10.1007/s10834-011-9274-z

[R23] Dwyer R. E. (2018). Credit, Debt, and Inequality. Annual Review of Sociology, 44(1), 237–261. 10.1146/annurev-soc-060116-053420

[R24] Eads A., & Tach L. (2016). Wealth and Inequality in the Stability of Romantic Relationships. RSF: The Russell Sage Foundation Journal of the Social Sciences, 2(6), 197–224. 10.7758/rsf.2016.2.6.10

[R25] Grall T. (2020). Custodial mothers and fathers and their child support: 2017, Current population reports (Nos. P60–269). U.S. Census Bureau, U.S. Department of Commerce, Economic and Statistics Administration. https://www.census.gov/library/publications/2020/demo/p60-269.html

[R26] Holden K. C., & Smock P. J. (1991). The economic costs of marital dissolution: Why do women bear a disproportionate cost? Annual Review of Sociology, 17, 51–78. 10.1146/annurev.so.17.080191.000411 12285404

[R27] Huang Y., Perales F., & Western M. (2019). To pool or not to pool? Trends and predictors of banking arrangements within Australian couples. PLOS ONE, 14(4), e0214019. 10.1371/journal.pone.0214019 30995218 PMC6469846

[R28] Kan M. Y., & Laurie H. (2014). Changing Patterns in the Allocation of Savings, Investments and Debts within Couple Relationships. The Sociological Review, 62(2), 335–358. 10.1111/1467-954X.12120

[R29] Kapelle N., & Baxter J. (2020). Marital dissolution and personal wealth: Examining gendered trends across the dissolution process. Journal of Marriage and Family, 83(1), 243–259. https://doi.org/10.1111/jomf.12707

[R30] Killewald A., Lee A., & England P. (2023). Wealth and Divorce. Demography, 60(1), 147–171. 10.1215/00703370-10413021 36661229

[R31] Kowalski R., Strzelecka A., Wałęga A., & Wałęga G. (2023). Do Children Matter to the Household Debt Burden? Journal of Family and Economic Issues, 44(4), 1007–1022. 10.1007/s10834-023-09887-y

[R32] Kreider R. M., & Ellis R. (2011). Number, Timing, and Duration of Marriages and Divorces: 2009 (Current Population Reports, pp. 70–125). U.S. Census Bureau. https://www2.census.gov/library/publications/2011/demo/p70-125.pdf

[R33] Leopold T. (2018). Gender differences in the consequences of divorce: A study of multiple outcomes. Demography, 55(3), 769–797. 10.1007/s13524-018-0667-6 29654601 PMC5992251

[R34] Leopold T., & Kalmijn M. (2024). Reassessing Chronic Strain: A Research Note on Women’s Income Dynamics After Divorce and Separation. Demography, 61(3), 597–613. 10.1215/00703370-11372303 38770913

[R35] Manning W. G., & Mullahy J. (2001). Estimating log models: To transform or not to transform? Journal of Health Economics, 20(4), 461–494. 10.1016/s0167-6296(01)00086-8 11469231

[R36] McCloud L., & Dwyer R. E. (2011). THE FRAGILE AMERICAN: Hardship and Financial Troubles in the 21st Century. The Sociological Quarterly, 52(1), 13–35. https://doi.org/http://www.jstor.org/stable/23027458.

[R37] McManus P. A., & Diprete T. A. (2001). Losers and winners: The financial consequences of separation and divorce for men. American Sociological Review, 66(2), 246–268. 10.2307/2657417

[R38] Mortelmans D. (2020). Economic consequences of divorce: A review. In Kreyenfeld M. & Trappe H. (Eds.), Parental Life Courses after Separation and Divorce in Europe (pp. 23–41). Springer International Publishing. 10.1007/978-3-030-44575-1_2

[R39] Munsch C. (2024). Household Debt Rose by $184 Billion in Q1 2024; Delinquency Transition Rates Increased Across All Debt Types. Federal Reserve Bank of New York. https://www.newyorkfed.org/newsevents/news/research/2024/20240514

[R40] Raley R. K., & Sweeney M. M. (2020). Divorce, Repartnering, and Stepfamilies: A Decade in Review. Journal of Marriage and Family, 82(1), 81–99. 10.1111/jomf.12651 38283127 PMC10817771

[R41] Rhodes A. P., Dwyer R. E., & Houle J. N. (2024). Debt Collection Pressure and Mental Health: Evidence from a Cohort of U.S. Young Adults. Journal of Health and Social Behavior, 00221465241268477. 10.1177/00221465241268477 PMC11867886 39225254

[R42] Rodems R., & Pfeffer F. T. (2021). Avoiding material hardship: The buffer function of wealth. Journal of European Social Policy, 31(5), 517–532. 10.1177/09589287211059043

[R43] Røed Bilgrei O., Alecu A. I., & Kok E. (2025). What are the Costs of Shared parenting? A Scoping Review. Family Transitions, 0(0), 1–22. 10.1080/28375300.2025.2596436

[R44] Smock P. J., Tzoc K., & Carr D. (2024). Gender and the Economic Consequences of Divorce in the United States: Variation by Race and Ethnicity. Journal of Family and Economic Issues, 45(4), 800–818. 10.1007/s10834-023-09940-w 41292516 PMC12643126

[R45] Sweet E. (2021). Debt-Related Financial Hardship and Health. Health Education & Behavior, 48(6), 885–891. 10.1177/1090198120976352 33241698

[R46] Thielemans G., & Mortelmans D. (2022). Poverty Risks after Relationship Dissolution and the Role of Children: A Contemporary Longitudinal Analysis of Seven OECD Countries. Social Sciences, 11(3), 138. 10.3390/socsci11030138

[R47] US Census Bureau. (2020). U.S. Marriage and Divorce Rates by State: 2008 & 2018. Census.Gov. https://www.census.gov/library/visualizations/interactive/marriage-divorce-rates-by-state-2008-2018.html

[R48] Act Wisconsin 186, Property Rights of Married Persons; Marital Property, 766.55 (1983).

[R49] Xiao J. J., & Yao R. (2020). Debt types and burdens by family structures. International Journal of Bank Marketing, 38(4), 867–888. (world). 10.1108/IJBM-07-2019-0262

